# Porous Semiconducting Polymer Nanoparticles as Intracellular Biophotonic Mediators to Modulate the Reactive Oxygen Species Balance

**DOI:** 10.1021/acs.nanolett.4c01195

**Published:** 2024-06-06

**Authors:** Miryam Criado-Gonzalez, Camilla Marzuoli, Luca Bondi, Edgar Gutierrez-Fernandez, Gabriele Tullii, Paola Lagonegro, Oihane Sanz, Tobias Cramer, Maria Rosa Antognazza, David Mecerreyes

**Affiliations:** † POLYMAT, University of the Basque Country UPV/EHU, Paseo Manuel de Lardizabal 3, 20018 Donostia-San Sebastián, Spain; ‡ Center for Nano Science and Technology@PoliMi, Istituto Italiano di Tecnologia, Via Raffaele Rubattino 81, 20134 Milano, Italy; § Politecnico di Milano, Dipartimento di Fisica, Piazza Leonardo da Vinci 32, 20133 Milano, Italy; ∥ Department of Physics and Astronomy, University of Bologna, Viale Carlo Berti Pichat 6/2, 40127 Bologna, Italy; ⊥ XMaS/BM28-ESRF, 71 Avenue Des Martyrs, F-38043 Grenoble Cedex, France; # Department of Physics, University of Warwick, Gibbet Hill Road, Coventry CV4 7AL, U.K.; 7 Department of Applied Chemistry, Faculty of Chemistry, University of the Basque Country UPV/EHU, Paseo Manuel de Lardizabal 3, 20018 Donostia-San Sebastián, Spain; 8 Ikerbasque, Basque Foundation for Science, 48013 Bilbao, Spain

**Keywords:** poly(3-hexylthiophene) semiconducting polymer, porous nanoparticles, organic electronics, reactive oxygen species (ROS), intracellular optical stimulation

## Abstract

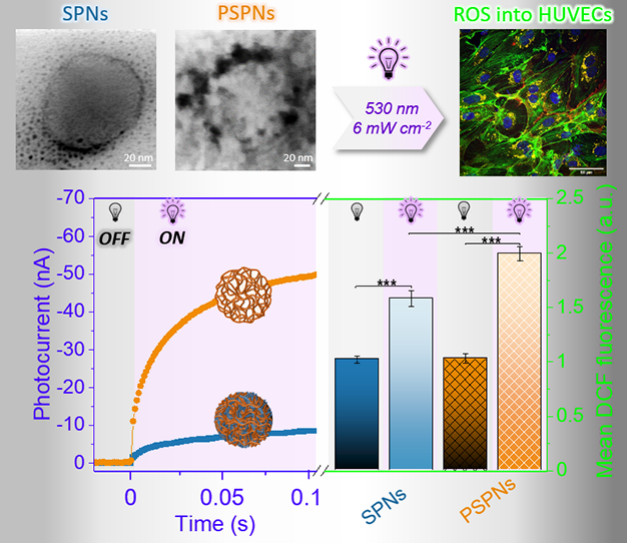

The integration of nanotechnology with photoredox medicine has led to the emergence of biocompatible semiconducting polymer nanoparticles (SPNs) for the optical modulation of intracellular reactive oxygen species (ROS). However, the need for efficient photoactive materials capable of finely controlling the intracellular redox status with high spatial resolution at a nontoxic light density is still largely unmet. Herein, highly photoelectrochemically efficient photoactive polymer beads are developed. The photoactive material/electrolyte interfacial area is maximized by designing porous semiconducting polymer nanoparticles (PSPNs). PSPNs are synthesized by selective hydrolysis of the polyester segments of nanoparticles made of poly(3-hexylthiophene)-*graft*-poly(lactic acid) (P3HT-*g*-PLA). The photocurrent of PSPNs is 4.5-fold higher than that of nonporous P3HT-*g*-PLA-SPNs, and PSPNs efficiently reduce oxygen in an aqueous environment. PSPNs are internalized within endothelial cells and optically trigger ROS generation with a >1.3-fold concentration increase with regard to nonporous P3HT-SPNs, at a light density as low as a few milliwatts per square centimeter, fully compatible with *in vivo*, chronic applications.

Reactive oxygen species (ROS) are biologically relevant oxidants generated in the human body that are of great interest in redox medicine as they can regulate many signal transduction pathways and cellular functions depending on their concentration.^1−4^ Regulation of vascular processes (i.e., angiogenesis) is essential for treating many diseases (e.g., cardiovascular pathologies and cancer) and intricately linked to the modulation of intracellular ROS levels.^5,6^ Whereas aberrant ROS production causes oxidative stress overload leading to cell dysfunction, inflammation, and tissue repair inhibition, a moderate increase in the level of ROS can regulate the fates of endothelial cells, promoting their proliferation and differentiation and the formation of new blood vessels fostering tissue regeneration.^7,8^

ROS can be generated endogenously, from the enzyme nicotinamide adenine dinucleotide phosphate oxidase, or exogenously through physical stimuli (i.e., light irradiation, electrical, thermal, or pharmaceutical methods).^9^ Compared to irreversible endogenous approaches and invasive electrical stimulus, optically controlled methods offer interesting prospects for wireless stimulation therapies.^10,11^ Nevertheless, the low efficacy due to the absorption of optical excitation by living tissues, the thermalization of the absorbed energy, and the variability of results observed across different cell and tissue models hamper their therapeutic application.^12,13^ Therefore, the development of photoactive materials capable of modulating ROS concentrations at nontoxic levels with low power densities in a reversible manner is a promising avenue for safer, gene-less, and minimally invasive optical modulation of the intracellular redox balance.

Semiconducting polymers (SPs) are attracting more attention as biophotonic materials because of their intrinsic conductivity, optical properties, biocompatibility, flexibility, and chemical versatility.^14−17^ Poly(3-hexylthiophene) (P3HT) is a highly biocompatible p-type polymer that has been employed for several applications in biophotonics, both in the form of thin films and as injectable beads.^18^ Interestingly, the ability to produce H_2_O_2_ and other intermediate ROS in aqueous media under aerobic conditions has recently been exploited to modulate the intracellular redox balance at nontoxic concentrations.^19−22^ Exogenous ROS production induced by light irradiation of P3HT films promotes the proliferation and formation of the tubular assembly in endothelial colony-forming cells.^23^ In addition, we recently demonstrated that the ROS production of P3HT films was also influenced by their structural engineering, where the optical absorption surface area available for the photoelectrochemical reactions greatly influenced the capability to efficiently reduce oxygen. The photon-to-ROS conversion yield of nanoporous P3HT films is higher than that of nonporous ones.^24^ However, the use of SP thin films for the precise modulation of the redox balance, while representing a useful test bed for *in vitro* studies, is certainly not ideal for practical *in vivo* applications due to numerous limitations. It requires surgical implantation. It provides only indirect redox balance modulation as it is active in the extracellular space. Its spatial selectivity is very limited as it cannot target specific cell subpopulations or intracellular compartments. It usually requires a high photoexcitation power density offering a limited dynamic range for fine-tuning ROS concentration, and the tissue inflammatory response may easily lead to a fast decline in its performance.^25^ A valuable solution is offered by SP nanoparticles (SPNs), which rapidly internalize within living cells, are prone to biochemical functionalization for selective cell targeting, and offer a fine ROS modulation capability in the intracellular compartment, thus potentially requiring a much lower power density for threshold activation and widening the range of available light energy density in a eustress regime.^26−29^ However, no attempts, to the best of our knowledge, to modify the morphology and form factor of plain SPNs have been reported so far, an approach that may instead reveal a key parameter for improving their versatility and photoelectrochemical efficiency at a lower power density, opening the way to unprecedented biotechnology applications in redox medicine.

Herein, we develop porous P3HT nanoparticles (PSPNs), characterized by an enlarged surface area, by nanoprecipitation followed by selective hydrolysis of the poly(lactic acid) (PLA) segments of a graft copolymer made of P3HT and PLA (P3HT-*g*-PLA). The physicochemical and structural properties of PSPNs were fully characterized, and the influence of the porosity on the photocurrent properties was explored. Finally, the use of PSPNs as intracellular wireless mediators for light-induced intracellular ROS production was tested in human umbilical vein endothelial cells (HUVECs), a biologically relevant model for the endothelium function whose working conditions are strictly governed by the intracellular ROS concentration and redox balance. Sizable enhancement of intracellular ROS concentration was achieved for P3HT-PSPNs (+100% vs control samples exposed to illumination, +30% vs nonporous P3HT-SPNs).

First, we investigated the synthesis of PSPNs using the selective hydrolysis method that involves the etching of one segment within a block or graft copolymer by taking advantage of the copolymer phase separation at the nanometer scale and the straightforward hydrolysis of polyester segments.^30−32^ Recently, we successfully employed this strategy to fabricate nanoporous thin films, used as extracellular tools for the effective modulation of the intracellular ROS concentration.^24^ Here, PSPNs were fabricated from graft copolymers, P3HT_*x*_-*g*-PLA_100–*x*_ (*x* = 28, 60 mol %) (Table S1), synthesized as previously reported. For the preparation of SPNs, graft copolymers P3HT_*x*_-*g*-PLA_100–*x*_ were solubilized in THF [90% (v/v)], and 10% (v/v) Pluronic F-127 (1 mg mL^–1^ in THF) was added as a biocompatible surfactant to guarantee the stabilization of the nanoparticles over time due to the steric, non-ionic repulsion between the shells around SPNs. Then, nonporous P3HT-*g*-PLA-SPNs were obtained by flash nanoprecipitation of P3HT_*x*_-*g*-PLA_100–*x*_/pluronic solutions in water under controlled conditions (Figure 1A). In a second step, porous nanoparticles (PSPNs) were obtained by selective hydrolysis of the PLA component of the graft copolymer in the presence of 1 M NaOH for 30 min, followed by a dialysis step to remove the byproducts.

**Figure 1:**
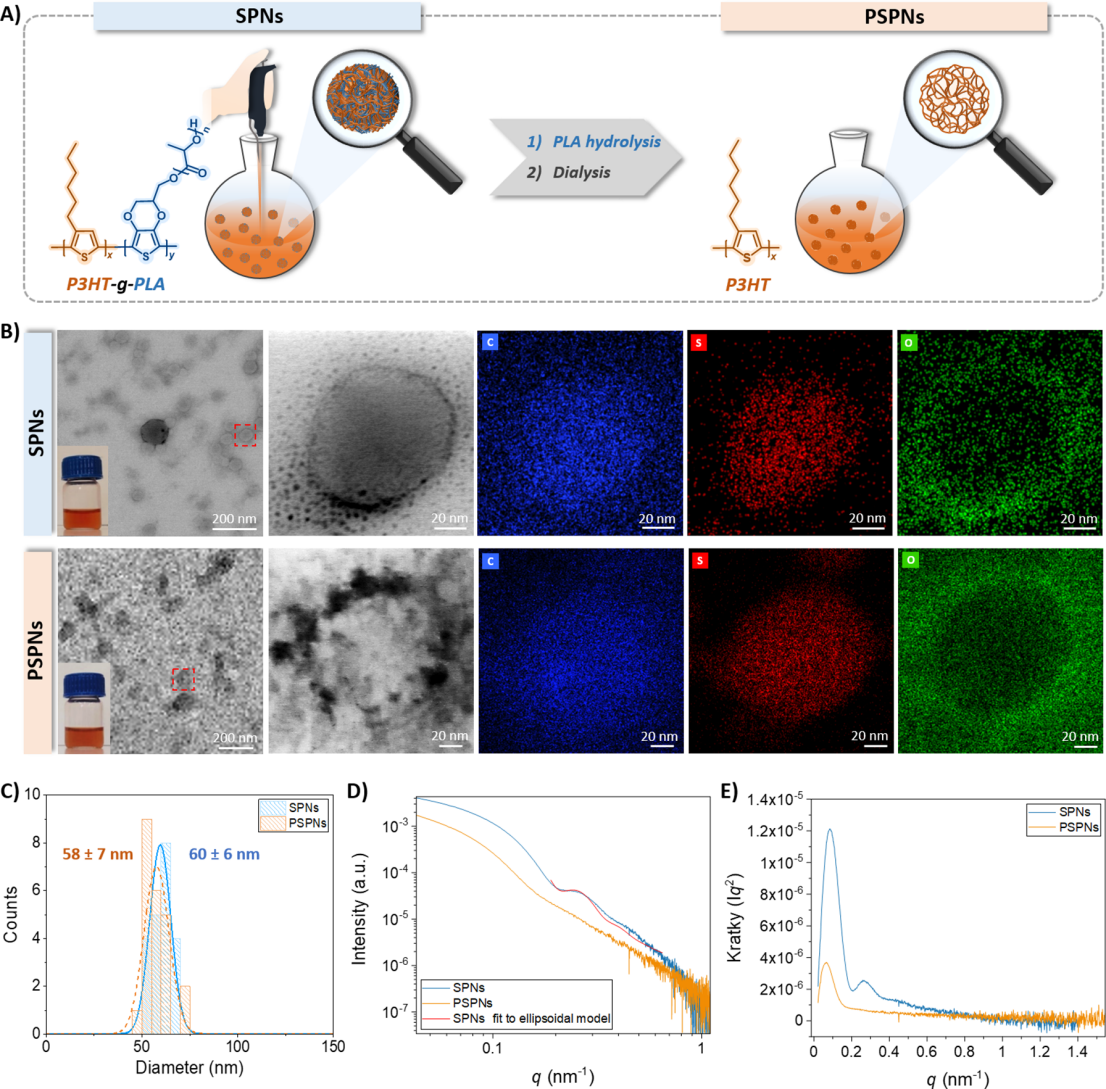
(A) Schematic representation of the synthesis of P3HT porous nanoparticles in two steps. Nonporous P3HT-*g*-PLA-SPNs were obtained by flash nanoprecipitation and subsequent PLA hydrolysis and dialysis to obtain porous P3HT PSPNs. (B) TEM images of SPNs and PSPNs. The insets show the photographs of the as-prepared nanoparticle dispersions, and dashed red squares highlight the areas selected for magnification of an isolated nanoparticle that was further analyzed by EDX. (C) Histograms of the dispersions of SPNs and PSPNs obtained from TEM images. (D) SAXS scattering curves of SPNs and PSPNs. (E) Kratky plots obtained from SAXS data.

Stable aqueous dispersions of SPNs and PSPNs were obtained from P3HT_60_-*g*-PLA_40_, and their morphology was analyzed by transmission electron microscopy (TEM) (Figure 1B). Nevertheless, PSPNs made of P3HT_28_-*g*-PLA_72_, with more PLA than P3HT in the graft copolymers, were disintegrated after PLA hydrolysis treatment and were not considered in subsequent experiments. SPNs displayed a round morphology with an average diameter of 60 ± 6 nm (Figure 1C). To delve into the distribution of both components, P3HT and PLA, in the SPNs, energy dispersive X-ray analysis (EDX) was performed. Sulfur atoms, which are present only in the P3HT segment of the copolymers, are colored red, and carbon atoms that are present in both segments, P3HT and PLA, are colored blue. The results exhibited a homogeneous distribution of both components throughout the nanoparticles. Oxygen atoms, present in high proportion in the pluronic, exhibited high intensity, forming a shell around the nanoparticles. Nanoparticles of the homopolymer P3HT (P3HT-SPNs) were synthesized as a control (Figure S1). After PLA hydrolysis, PSPNs retained an approximately round morphology and a comparable diameter of 58 ± 7 nm (Figure 1B,C). EDX analysis allowed us to corroborate that the red color of sulfur atoms present in the P3HT remained stable, keeping the same distribution as in SPNs, whereas the blue color intensity of carbon atoms decreased as the PLA domains disappeared. The oxygen atoms of the pluronic (green) also formed a stable shell around the nanoparticles. The ζ potential of the nanoparticles was determined by dynamic light scattering (DLS). SPNs and PSPNs exhibited similar ζ values, −23.6 ± 1.1 and −22.5 ± 1.0 mV, respectively, as expected because of the presence of sulfate atoms of P3HT on their surface not fully screened by pluronic. These values are also in agreement with that obtained for P3HT-SPNs (ζ = −23.4 ± 1.1 mV). Negatively charged nanoparticles are preferred due to their better stability and weaker tendency to agglomerate.^33^ The chemical composition was analyzed by proton nuclear magnetic resonance (^1^H NMR). The spectrum of the SPNs exhibited the characteristic peaks of P3HT at 0.9 ppm and PLA at 5.2 ppm, together with the pluronic signals at 2.2 and 3.7 ppm.^34−36^ After hydrolysis treatment, the spectrum of the PSPNs showed only the characteristic peaks of P3HT and pluronic, whereas no PLA signal was detected, meaning that PLA was totally hydrolyzed inducing the porosity of the nanoparticles (Figure S2). TEM images confirmed the round shape of the nanoparticles, their unaltered size after PLA hydrolysis, and their component distribution. However, they could not provide information about the inner structure of the nanoparticles, the pore size, or their distribution. Therefore, small angle X-ray scattering (SAXS) measurements were performed. The SAXS profile of SPNs showed two distinct peaks attributed to the inherent nanoparticle form factor (Figure 1D). Fitting the data in the peak region allowed us to extract quantitative insights into the shape and size of the nanoparticles in response to X-rays. For this fitting, a polydisperse ellipsoidal model was employed with a polar diameter of 50 nm and an equatorial diameter of 40 nm, both with a polydispersity index set at 0.15. The SAXS profile of PSPNs did not show any recognizable form factor. This became even clearer when comparing the Kratky plots (Figure 1E). No distinctive features of SPNs disappeared after porosity was induced. The Kratky plot shape for PSPNs closely resembled those observed for globular folded proteins, characterized by an irregular and highly textured surface that lacks the regular features that would contribute to a recognizable form factor.^37^ This implied that the hydrolysis procedure affected the entire particle rather than just the surface, resulting in an irregularly porous P3HT bead with a substantial density of holes and empty spaces. Consequently, PSPNs exhibited a larger surface area as determined by N_2_ gas adsorption–desorption measurements. The Brunauer–Emmet–Teller (BET) surface areas of SPNs and PSPNs were 1.2 and 64.5 m^2^ g^–1^, respectively, providing a 54-fold increase in the surface area of PSPNs compared to that of SPNs. This was supported by TEM images revealing an exceptionally irregular shape (Figure 1C).

Ultraviolet–visible spectra of both SPNs and PSPNs (Figure 2A) exhibited a prominent absorption peak at 480 nm, attributed to a flexible random-coil conformation of the P3HT chains. The peak at 593 nm is indicative of P3HT interchain interactions promoting a higher degree of order.^38^ The similarity in both spectra indicates that porosity does not preclude their use in optoceutical therapies.^39,40^ Both fluorescence spectra (Figure 2B) were apparently similar, with a maximum emission peak at 634 nm, from the 0–0 transition, and a shoulder at 710 nm, from the 0–1 vibronic transition;^41^ however, some differences appeared in the exciton bandwidth as explained in detail in Table S2.^42,43^ These results proved the occurrence of structural changes in PSPNs with respect to SPNs, while confirming their main optical responsivity in the visible range.

**Figure 2:**
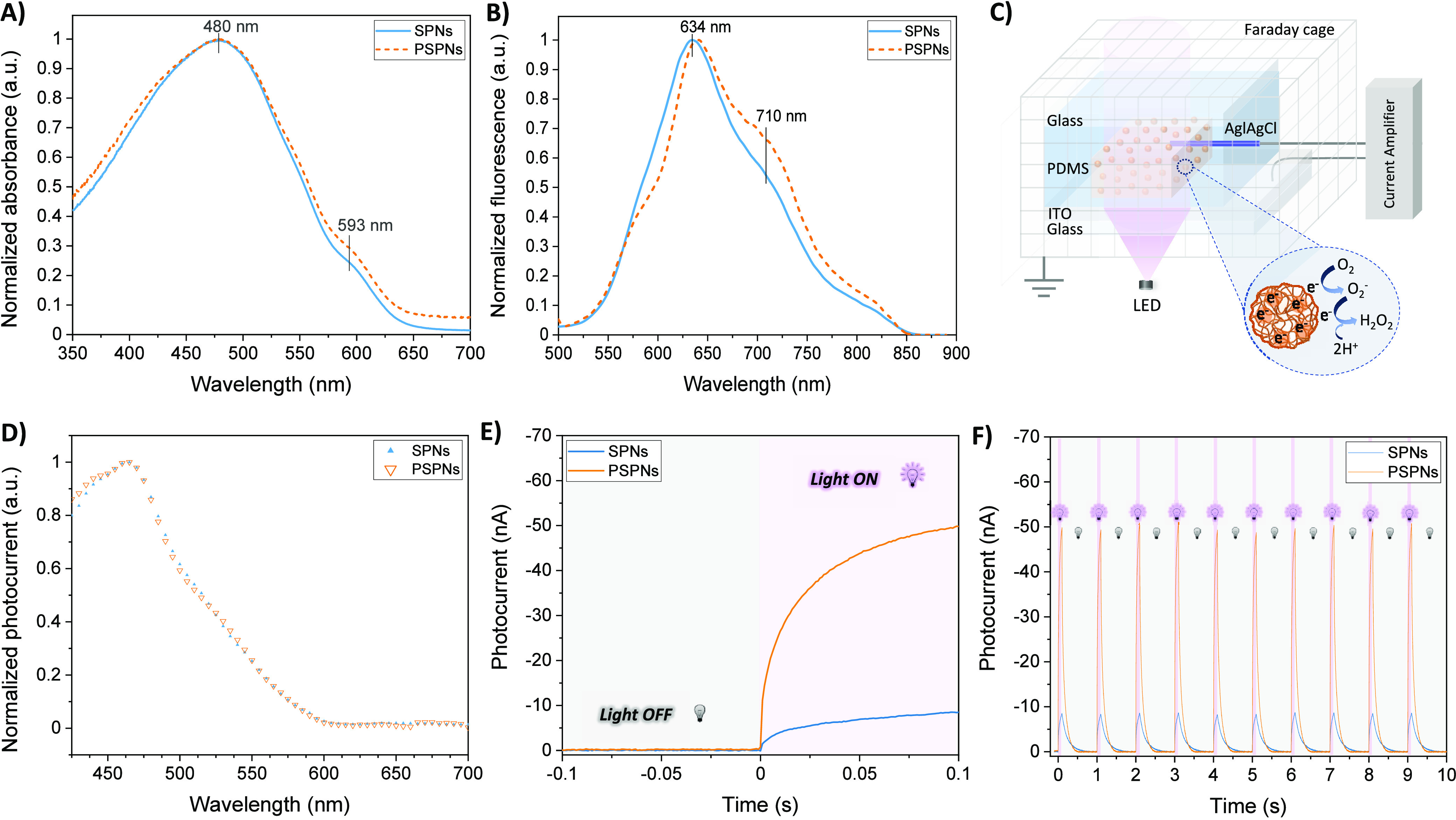
(A) Normalized optical absorption spectra of SPNs and PSPNs. (B) Normalized fluorescence spectra (λ_exc_ = 480 nm) of SPNs and PSPNs. (C) Schematic representation of the photoelectrochemical cell (PEC) used for measuring the photocurrent properties, including oxygen reduction reactions following nanoparticle irradiation. (D) Normalized photoelectrochemical current spectra of SPNs and PSPNs in contact with the electrolyte (0.1 M PBS at pH 7.4). (E) Photocurrent curves of SPNs and PSPNs upon their irradiation with a LED (λ = 530 nm; 6 mW cm^–2^). (F) Photocurrent curves of the initial 10 on–off cycles of SPNs and PSPNs in the dark and upon irradiation with a LED (λ = 530 nm; 6 mW cm^–2^; 100 ms on and 900 ms off) mimicking cellular stimulation conditions.

The photoelectrochemical properties were determined by using a transparent ITO electrode as the working electrode in contact with a dispersion of nanoparticles in an aqueous electrolyte [0.1 M phosphate-buffered saline (PBS) (pH 7.4)]. A chlorinated silver wire was used as a pseudoreference electrode, and a Faraday cage was used to shield external noises (Figure 2C). A photocathodic current was produced upon illumination through the electrolyte, as photoexcited electrons were transferred from the SPNs to acceptor states in the electrolyte such as dissolved oxygen and positive charge accumulated in the SPNs. Metastable O_2_^–^ is expected to undergo a rapid dismutation leading to H_2_O_2_ formation. Subsequently, SPNs diffused and ultimately discharged at the ITO electrode, producing the measured current signal. As the concentration of the SPNs and other parameters were held constant, the resulting current could be correlated with the photoreduction efficiency of the nanoparticles, and the spectral response could be characterized. Normalized photocurrent spectra were identical for both SPNs and PSPNs showing a maximum at 470 nm (Figure 2D), supporting the hypothesis of no substantial structural differences introduced into the semiconductor structure by the porosity. To study the evolution of the photocurrent over time, samples were irradiated at a fixed wavelength with a blue light-emitting diode (LED) (λ = 530 nm). The irradiation promptly induced the generation of a cathodic photocurrent (<50 ms) due to the oxygen reduction reactions,^25,26^ achieving a steady state after 100 ms (Figure 2E). A 4.5-fold increase in photocurrent was observed for PSPNs compared to that of SPNs. Such a high activity of PSPNs enables *in vitro* cell testing at much lower light intensities than those for SPNs, moving conditions closer to the needs for clinical applications. Then, to mimic the photostimulation conditions of SPNs in contact with living cells, we measured photocurrent transients generated by low-intensity light pulses (6 mW cm^–2^, 100 ms duration) (Figure 2F). The results followed the same trend as in the previous experiments, achieving a 4.5-fold increase in the photocurrent for PSPNs compared to that for SPNs and a perfect cyclic behavior during subsequent 100 ms on – 900 ms off irradiation steps. After completion of the 6 h irradiation cycle, the photocurrent yield slightly decreased by ∼15% (Figure S3).

The regulation of cell fate, encompassing adhesion, proliferation, and migration, is strictly related to the intracellular redox state and the capacity to generate or quench ROS.^44^ We studied the impact of the nanoporous morphology of polymer beads on the photoinduced modulation of intracellular ROS production using HUVECs as a relevant biological model to study the endothelium function. Cell proliferation of HUVECs treated with SPNs and PSPNs at different concentrations (10 and 30 μg mL^–1^) was evaluated by the Alamar Blue assay (Figure S4). No cytotoxic effects on the proliferation of HUVECs were observed for either SPNs or PSPNs up to 120 h, and no significant differences were observed between the two concentrations. Therefore, we chose an intermediate concentration (20 μg mL^–1^) for further *in vitro* cell experiments and included the P3HT-SPNs in the analysis, as a control displaying a nonporous morphology along with the same chemical structure of PSPNs. We evaluated the nanoparticle distribution within the cells by confocal fluorescence microscopy, in both live and fixed cells (Figure S5 and Figure 3A, respectively). In the second case, HUVECs were stained with DAPI (cell nuclei, blue fluorescence) and with phalloidin (cytoskeleton, green fluorescence). Full z-stacks (Figure S6) and representative images, acquired at the z-plane corresponding to the intracellular space (Figure 3A), showed that each type of nanoparticle (intrinsic fluorescence, colored yellow) was successfully internalized within the cells and localized in the perinuclear region. We also employed the antigen CD31 (red fluorescence), which is strongly expressed in endothelial cells.^29^ The impact of light irradiation (λ = 530 nm; 6 mW cm^–2^; 100 ms on and 900 ms off, for 6 h) on cell viability and proliferation over time was evaluated (Figure 3B). Nonsignificant differences were observed in cell proliferation between the control (CTRL, cells seeded on glass substrates without SPNs) and the considered conditions (P3HT-SPNs, P3HT-*g*-PLA-SPNs, and P3HT-PSPNs), in the dark or upon illumination. Finally, we investigated intracellular ROS production through fluorescence microscopy by incubating HUVECs with the 2′,7′-dichlorodihydrofluorescein diacetate (H2-DCF-DA) probe (Figure 3C). This probe is sensitive to a large variety of ROS, including short-lived species (O_2_^–^, OH^–^, and ONOO^–^; lifetime of <0.1s) and long-lived ones (H_2_O_2_). However, we should consider that under our conditions (elapsed time between photostimulation and ROS measurement of ≫0.1 s), only light-induced long-lived species are expected to contribute to the recorded signal. Data were normalized to the control (CTRL). Under the dark conditions, all samples exhibited a similar ROS production activity, as expected. Upon illumination, P3HT-SPNs showed a statistically significant increase in the level of intracellular ROS production, in agreement with previous reports.^26,45^ On the contrary, no increase was noted for P3HT-*g*-PLA-SPNs, which might be attributed to the lower photocurrent intensity exhibited by P3HT-*g*-PLA-SPNs than by pure P3HT-SPNs. Remarkably, P3HT-PSPNs exhibited a >1.3-fold increase in the level of ROS production upon illumination, thereby confirming the close relationship among the porosity of PSPNs, the photocurrent density, and the modulation of intracellular ROS production. Recently, porous thin films were similarly reported to induce an increase in intracellular ROS concentration.^24^ However, it is important to underline that the results presented here represent an important step forward, toward the possibility of employing SPNs for selective optical modulation of the cell redox balance. The successful synthesis of PSPNs enables important advantages that cannot be achieved in the case of SP thin films. (i) PSPNs efficiently internalize within the cell cytosol, constituting an intracellular source of ROS at variance with SP thin films. Selective targeting of specific cell subpopulations, or even cell organelles, could be conceivable upon proper functionalization of PSPNs using standard protocols reported for organic polymers such as linking with specific antibodies or aptamers. (ii) The porous morphology represents a favorable form factor for a more intimate biointerface with cells, a key step in effective modulation of cell homeostasis. (iii) The use of more efficient porous PSPNs within the intracellular space allows for a sizable decrease in the light power density, as compared to that of nonporous SPNs. This allows ad hoc modulation of the intracellular ROS concentration over different orders of magnitude to enable use in either eustress, mild distress, or severe stress conditions, according to the therapeutic need.

**Figure 3:**
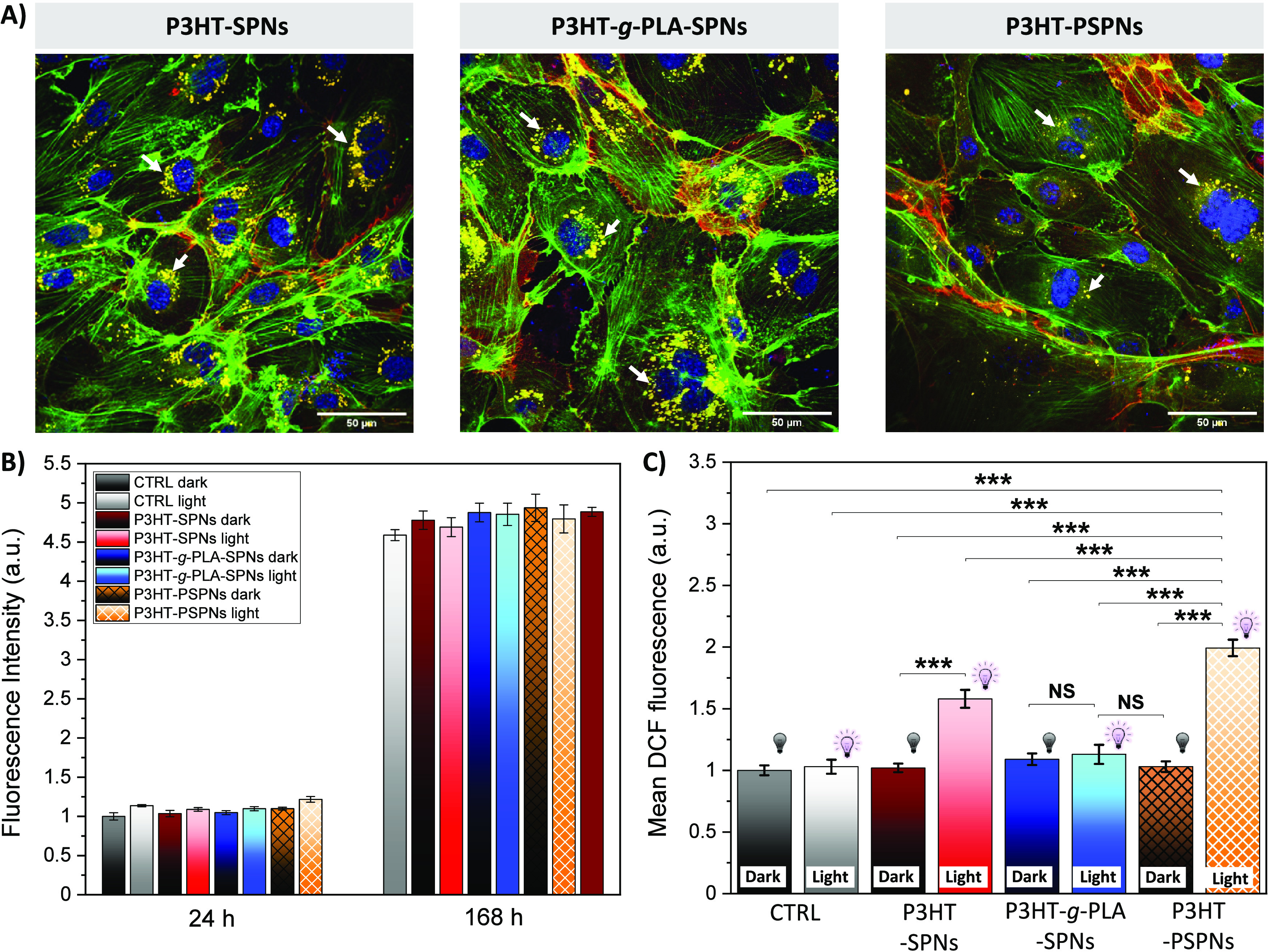
(A) Representative confocal fluorescence images of HUVECs treated with P3HT-SPNs, P3HT-*g*-PLA-SPNs, and P3HT-PSPNs. Immunofluorescence images show nuclei (blue), actin filaments (green), and CD31 (red). Nanoparticles are visible because of their intrinsic fluorescence (represented in yellow for better visualization). The scale bars are 50 μm. (B) *In vitro* proliferation assay of HUVECs, nontreated (CTRL) and in contact with SPNs (solid bars) and PSPNs (frame filler bars) up to 168 h. (C) *In vitro* intracellular ROS production evaluated as the fluorescence of the H2-DCF-DA probe. HUVECs treated with SPNs (solid bars) and PSPNs (frame filler bars) under both dark and light conditions (LED centered around λ = 530 nm, power density of 6 mW cm^–2^, 100 ms on and 900 ms off for 6 h). The results are shown as the mean ± standard error of the mean (three biological replicates). Analysis of variance (ANOVA) statistical test, Bonferroni correction at significance levels of ***p < 0.001, **p < 0.01, and *p < 0.05.

In summary, P3HT-PSPNs were designed as efficient intracellular mediators for light-activated ROS generation allowing spatiotemporally resolved intracellular photoelectrochemical ROS generation without applying any bias and without resorting to gene-cell modification. PSPNs were synthesized by flash nanoprecipitation of a hydrolyzable p-type semiconducting graft copolymer, P3HT_60_-*g*-PLA_40_, followed by selective PLA graft hydrolysis. P3HT-PSPNs displayed an average diameter of ∼60 nm and a ζ of approximately −23 mV. SAXS measurements revealed that the structure of P3HT-PSPNs resulted in a porous P3HT bead with a substantial density of holes and empty spaces with a larger active surface area. P3HT-PSPNs demonstrated excellent responsiveness to low-power light (λ = 530 nm; 6 mW cm^–2^), showcasing a 4.5-fold increase in photocurrent properties compared to those of nonporous SPNs. P3HT-PSPNs were internalized in HUVECs without inducing cytotoxic effects. Furthermore, P3HT-PSPNs induced a >1.3-fold increase in the level of ROS production upon illumination under mild conditions akin to clinical applications. To the best of our knowledge, we have reported the first example of cytocompatible porous SPNs, which presents distinct advantages over the current state of the art. (i) Compared to porous SP thin films, PSPNs can finely modulate the ROS concentration in an intracellular manner because of their fast internalization within the cell cytosol. (ii) Compared to other SPNs, the strategy adopted here of increasing the surface/volume ratio provided higher photoelectrochemical efficiency toward oxygen reduction, making it possible to sizably reduce the light power density. (iii) Compared to other nanostructured inorganic materials used in biophotonic applications, PSPNs possessed excellent photostability and cytocompatibility to work in an eustress regime, with a high level of interest in the emerging field of photoredox medicine. Overall, P3HT-PSPNs are promising tools for highly efficient, touchless, and spatiotemporally resolved ROS modulation in vascular tissue.

## Supporting Information Available

Materials and methods, characterization data of P3HT and P3HT-*g*-PLA, peak intensity ratio from the absorption and emission spectra of SPNs, TEM images of P3HT-SPNs, ^1^H NMR spectra of P3HT-*g*-PLA-SPNs before and after hydrolysis, cyclic photocurrent curves of SPNs and PSPNs, Alamar Blue assays of SPNs, and confocal fluorescence microscopy images of SPNs internalized in HUVECs (PDF)
