# Supplementary material for: Porous Semiconducting Polymer Nanoparticles as Intracellular Biophotonic Mediators to Modulate the Reactive Oxygen Species Balance
Source: Nano Lett. 2024 Jun 6;24(24):7244–51. doi: 10.1021/acs.nanolett.4c01195 (PMC11194851; doi:10.1021/acs.nanolett.4c01195)
Supplement: Supplementary file 1 — nl4c01195_si_001.pdf [file nl4c01195_si_001.pdf]

Supporting Information

Porous Semiconducting Polymer Nanoparticles as Intracellular Biophotonic Mediators to Modulate the Reactive Oxygen Species Balance

Miryam Criado-Gonzalez,[1] Camilla Marzuoli,[2,3] Luca Bondi,[4] Edgar Gutierrez-Fernandez,[1,5,6] Gabriele Tullii,[2] Paola Lagonegro,[2] Oihane Sanz,[7] Tobias Cramer,[4] Maria Rosa Antognazza,[2] and David Mecerreyes[1,8]**

[1] POLYMAT, University of the Basque Country UPV/EHU, Paseo Manuel de Lardizabal 3, 20018 Donostia-San Sebastián, Spain

[2] Center for Nano Science and Technology@PoliMi, Istituto Italiano di Tecnologia, Via Raffaele Rubattino 81, 20134 Milano, Italy

[3] Politecnico di Milano, Dipartimento di Fisica, Piazza Leonardo da Vinci 32, 20133 Milano, Italy

[4] Department of Physics and Astronomy, University of Bologna, Viale Carlo Berti Pichat 6/2, 40127 Bologna, Italy

[5] XMaS/BM28-ESRF, 71 Avenue Des Martyrs, F-38043 Grenoble Cedex, France

[6] Department of Physics, University of Warwick, Gibbet Hill Road, Coventry, CV4 7AL, UK

[7] Department of Applied Chemistry, Faculty of Chemistry, University of the Basque Country UPV/EHU, Paseo Manuel de Lardizabal 3, 20018 Donostia-San Sebastián, Spain

[8] Ikerbasque, Basque Foundation for Science, 48013 Bilbao, Spain

Materials and methods

Materials: 3-hexylthiophene ≥ 98.0% (3HT) and hydroxymethyl EDOT ≥ 97.0% were supplied by TCI, iron (III) chloride, sodium hydroxide, and methanol ≥ 99.9% were purchased from fluka, methanesulfonic acid ≥ 99.0% (MSA), and 4-(dimethylamino)pyridine ≥ 98.0% (DMAP) were provided by Sigma-Aldrich and used as received. L-lactide ≥ 85.0% (LA) was purchased from Sigma-Aldrich and purified by solving the lactic acid in toluene at 60 ºC followed by the precipitation and crystallization at room temperature twice. Then, the toluene was removed by rotary evaporation and the final product was dried under vacuum. The rest of the chemicals were used as received.

Synthesis of P3HT and P3HT-g-PLA copolymers: P3HT was synthesized by chemical oxidative copolymerization of 3-hexylthiophene using $FeCl_3$ as an oxidizing agent (4 eq. respect 3HT monomer) and acetonitrile as solvent at room temperature overnight. Graft copolymers with different compositions were synthesized by chemical oxidative copolymerization of 3-hexylthiophene and an α-EDOT-PLA macromonomer using $FeCl_3$ (4 eq. respect 3HT monomer) as oxidizing agent and acetonitrile as solvent as reported previously[24] (Table S1).

Nanoparticles synthesis: semiconducting polymer nanoparticles (SPNs) were obtained by flash nanoprecipitation. To that aim, the homopolymer P3HT or copolymer P3HT-*g*-PLA was solubilized at a concentration of 1 mg mL^{-1} in tetrahydrofuran (THF), and using Pluronic F-127 (1 mg mL^{-1} in THF) as surfactant in a ratio (%v/v) polymer:pluronic of 9:1, by stirring at 60 ºC for 1 h. Then, 1 mL of the polymer:pluronic solution was injected into 10 mL of Milli-Q water under stirring at 1300 rpm and 85 ºC. After 10 seconds, the nanoparticles dispersion was transferred to a beaker to evaporate the THF at 50 ºC under stirring for 90 minutes. Porous nanoparticles (PSPNs) were obtained in a second step by PLA hydrolysis in 1M NaOH for 30 minutes, followed by a dialysis purification step in Milli-Q water for 3 days. Finally, the SPNs dispersion was centrifuged at 500 rpm for 10 minutes to separate macroscopic aggregates.

Proton nuclear Magnetic Resonance spectroscopy (^{1}H-NMR): ^{1}H-NMR spectra were recorded with a Bruker Advance DPX 300 at 300.16 MHz resonance frequency, room temperature, and

using $CDCl_3$ as solvent. The experimental conditions were: 10 mg of sample, 3 s acquisition time, 1 s delay time, 8.5 μs pulse, spectral width 5000 Hz, and 32 scans.

Transmission Electron Microscopy (TEM) and Energy Dispersion X-ray Analysis (EDX): The structure and morphology of non-porous and porous films were visualized by TEM using a Talos F200i field emission gun instrument equipped with a Bruker X-Flash100 XEDS spectrometer. Nanoparticles dispersions were deposited over carbon-coated copper grids, and dried at room temperature before observation. Elemental maps were performed by XEDS in the STEM mode under a high annular dark field (HAADF) detector for Z contrast imaging in STEM conditions (camera length of 160 mm) using a pixel size of 28 nm, a dwell time of 900 s and an image size of 512 × 512 pixels. Moreover, EDX microanalyses were carried out using a probe current of 500 pA and a semiconvergence angle of 8.5 mrad. TEM images were analyzed with the software ImageJ.

Dynamic Light Scattering (DLS): DLS measurements were performed with a Malvern Nanosizer NanoZS Instrument equipped with a 4 mW He-Ne laser (λ = 633 nm) at a scattering angle of 173° and 25°C. Three measurements of 20 runs were performed for each sample.

Small Angle X-ray Scattering (SAXS): SAXS experiments were performed at NCD-SWEET beamline (BL-11), at ALBA Synchrotron (Barcelona, Spain), using an X-ray wavelength of λ = 0.1 nm. Nanoparticle dispersions (in water) were poured inside borosilicate glass capillaries (1.5 mm diameter and 0.01 mm wall thickness from WJM-Glas Müller GmbH). The samples were measured in transmission mode, exposing every sample to the beam for 10-20 seconds. Due to the low concentration of the samples, we had to increase the statistics by collecting up to 10 frames. The energy beam was set to 12.4 keV. The patterns were collected using a Pilatus3 S 1M detector, by Dectris, located at 6.70 m from the sample position. The SAXS patterns were normalized using a monitor value and reduced by azimuthal integration in all polar directions. The resulting intensity profiles were corrected by subtracting the scattering profile from the same capillary filled with water. To fit the SAXS profile, we used SasView software (http://www.sasview.org/).

Nitrogen adsorption-desorption. The specific surface area of the nanoparticles was characterized by N_2 adsorption–desorption at 77 K in a Micromeritics ASAP 2020. Prior to the measurements, the samples were degassed at 303 K for 48 h under vacuum. From N_2 adsorption–desorption

isotherms, the specific surface area was calculated from the Brunauer–Emmett–Teller (BET) equation.

UV-vis and Fluorescence Spectroscopies: UV-vis spectra were recorded from 350 to 700 nm with a Shimadzu UV-2550 spectrometer. Fluorescence emission spectra were recorded from 500 to 900 nm, after exciting the samples at a wavelength of 480 nm, with a Perkin Elmer LS 55 Fluorescence Spectrometer.

Photocurrent spectroscopy: For spectroscopic measurements, monochromatic light was produced using a 250 W xenon light source and a Cornerstone 260 monochromator with a 2 mm entrance slit and a 2 mm exit slit. The light was modulated at 7 Hz using a rotating disc chopper. To prevent second harmonics, a 400 nm long pass filter was inserted into the beam for measurements with wavelengths greater than 400 nm. The measurement cell, positioned 70 mm from the monochromator exit slit, yielded an average light intensity of 219 ± 8 μW cm^{-2} in the 400-800 nm range. The measurement cell contained a PDMS liquid reservoir with a nanoparticle suspension (approximately 200 μL), which was illuminated through a glass window and in contact with an ITO-covered glass slide serving as the working electrode. An Ag|AgCl wire functioned as a quasi-reference/counter electrode. The measurement cell was surrounded by a grounded aluminum structure acted as mechanical support and Faraday cage. For transient measurements the monochromated Xenon lamp light was replaced with monochromatic LEDs (Thorlabs M530L4) driven by a source-measure unit (ThorlabsDC2200). The photocurrent signal was detected, and the potential applied to the working electrode was controlled using a current amplifier (FEMTO-DLPCA). The amplified signal was subsequently acquired using the lock-in function for spectral acquisitions, and the oscilloscope or plotter functions, based on the timescales, for transient acquisitions. The instrument utilized was a multi-purpose lock-in amplifier (ZHINST, MFLI). Spectra were appropriately normalized by considering the detector spectral responsivity, the emission spectrum of the light source, and the spectral efficiency curves of the monochromator's gratings.

HUVECs cell culture and plating: HUVECs were purchased from PromoCell and grown in endothelial cell basal medium (PromoCell), supplemented with Endothelial cell GM 2 supplement pack (PromoCell). Cells were cultured in T-75 culture flasks pre-treated with 0.2% gelatin and

maintained in incubator at 37 °C in a humidified atmosphere with 5% CO_2. For the experiments, only HUVECs at passage < 7 were employed. After reaching 80–90% confluence, cells were enzymatically detached by incubation with 0.5% trypsin-0.2% EDTA (Sigma Aldrich) for 5 min. Prior to cell plating, a layer of 1 mg mL^{-1} fibronectin (from bovine plasma, Sigma Aldrich) in phosphate buffer saline (PBS, Sigma Aldrich) was deposited on the surface of the samples and incubated for at 37 ºC for 30 min, to promote cell adhesion. Excess fibronectin was then removed, and cells were plated on glass samples. NPs were added to the cell culture 3h after plating. Chronic stimulations were carried for 6 hours inside of the incubator with LEDs (emission wavelength peaked at 530 nm), employing a pulsed protocol (λ = 530 nm; 6 mW cm^{-2}, 100 ms ON and 900 ms OFF) for 6 h.

AlamarBlue viability assay: HUVECs were plated at low density (about 1×10^4 cells cm^{-2}) directly in fibronectin-treated 96 well. 3 h after plating, cells were treated with NPs at different concentrations and stimulation of NPs was performed overnight by employing a chronic stimulation protocol (λ = 530 nm; 6 mW cm^{-2}, 100 ms ON and 900 ms OFF) for 6 h. The metabolic activity of HUVECs was assessed by the AlamarBlue (ThermoFisher Scientific, catalog number DAL1100) fluorescence assay. Quantification of cell proliferation is based on the compound resazurin, which is able to permeate the cell membrane and is irreversibly reduced by the mitochondrial respiratory chain of cells to the highly fluorescent compound resorufin. Cell proliferation was evaluated by incubating the AlamarBlue reagent for 3 h at 37 ºC, 5% CO_2 in dark conditions. Three aliquots of culture medium containing the AlamarBlue reagent were placed in black 96-well microplates. Using a Tecan Spark microplates reader it was possible to measure the fluorescence intensity of resorufin (excitation wavelength: 530 nm, emission wavelength: 590 nm), which is directly proportional to the amount of metabolically viable cells. The cell culture medium was then replaced, allowing the proliferation of the same cell culture to be followed over time. Results are averaged over three instrumental replicates for each condition and normalized over the CTRL condition of the first time point. Measurements were then repeated in three biological replicates and averaged.

Confocal Imaging by Immunofluorescence Microscopy: HUVECs were seeded on 18mm glass coverslip at a density of 1×10^4 cells cm^{-2} and treated with NPs 3 h after plating. 20 h after nanoparticles addition the cells were washed with Krebs Ringer's (KRH) extracellular solution

(mM): 135 NaCl, 5.4 KCl, 1.8 $CaCl_2$, 1 $MgCl_2$, 5 HEPES, 10 Glucose, pH adjusted to 7.4 with NaOH. Then the cells were directly prepared for live confocal imaging or fixed with paraformaldehyde. In the former case, the cells were stained with Cell Mask Green (cell membrane, Thermo Fisher, exc/em wavelength, 522/535 nm) and HOECHST (nuclei, Thermo Fisher, exc/em, 350/461 nm). Z-stacks were acquired with an upright microscope (Olympus BX63), equipped with a spinning disk confocal module (X-Light V2 spinning disk module from Crest Optics), a 60X water immersion objective, and a sCMOS Camera (Prime BSI, Teledyne Photometrics; Tucson, Arizona, USA). The system was assembled by Crisel Instruments. In the latter case, the cells were washed with PBS, fixed with 4% paraformaldehyde for 10 min and washed three times with PBS. HUVECs were incubated for 45 min with blocking buffer (PBS + 0.05% v/v Tween 80 + 0.5% v/v FBS (fetal bovin serum) to reduce nonspecific staining. After removal of the blocking buffer, cells were incubated with phalloidin 1:1000 in blocking solution for 5 min and washed three times with PBST (PBS + 0.05% v/v Tween 80) to stain the actin filament. For membrane staining, cells were soaked overnight at 4 °C with a Purified Anti-Human CD31 in mouse Antibody (Biolegend, USA) diluted in blocking buffer, respectively. Cells were then washed three times with PBST and incubated for 4 h at room temperature with mouse secondary antibody (FAB2 ANTI-MO-IGG POLYEF 660 red mouse 1:500, Thermofisher, USA). Cells were then washed three times with PBST and stained with DAPI solution (1:500) in PBS to the color the nuclei. Finally, samples were mounted on microscope slides with Moviol and cell fluorescence was captured using a Nikon A1 Confocal Microscope. Since the fluorescence emission of the secondary antibody FAB2 lies within a wavelength range close to the one of nanoparticles intrinsic emission, we acquired the confocal images in the spectral mode to better distinguish them. Then, for a better visualization, we employed red color for the membrane, and yellow false color for nanoparticles. Images were analyzed using Fiji ImageJ software.

Intracellular ROS detection: Intracellular ROS were detected by employing 2',7'-dichlorodihydrofluorescein diacetate (H2-DCF-DA, purchased from Sigma Aldrich), sensitive to a large variety of ROS. The non-fluorescent H2-DCF-DA is internalized within cells, where intracellular esterases remove the acetate groups hydrolyzing H2-DCF-DA to H2-DCF. This molecule is then oxidized by ROS present in the cytosol to produce the highly fluorescent compound 2,7-dichlorofluorescein (DCF). For intracellular ROS detection, HUVECs were plated directly on a 96-well pre-treated with fibronectin. Cells were plated at 1×10^4 cells cm^{-2} density

and treated with nanoparticles 3 h after plating. 15 h after plating, the cells were subjected to the chronic illumination protocol (λ = 530 nm; 6 mW cm^{-2}, 100 ms ON and 900 ms OFF) for 6 h. Then, the cells were, incubated with H2-DCF-DA for 30 min, washed with KRH solution, and the DCF fluorescence was acquired with Nikon Ti inverted microscope, equipped with a 20X objective and a sCMOS Camera (Prime BSI, Teledyne Photometrics; Tucson, Arizona, USA). Results are averaged over three instrumental replicates for each condition and normalized over the CTRL condition. Measurements were then repeated in three biological replicates, significance was evaluated by ANOVA statistical test, Bonferroni correction at a significance level of $*p < 0.05$.

Supplementary Tables and Figures

Table S1. Characterization data of the homopolymer P3HT and graft copolymers P3HT-*g*-PLA by SEC and ^{1}H NMR.

Sample[a]	M_n (g mol^{-1})[b]	D[c]	RR[d] (%)
P3HT	17,400	2.01	75
$P3HT_{60}$-*g*-PLA_{40}	16,300	2.69	74
$P3HT_{28}$-*g*-PLA_{72}	24,000	1.80	76

[a] Mol fraction of P3HT and PLA in the copolymers calculated by ^{1}H NMR.
[b] M_n determined by using PS standards.
[c] Dispersity (D) = M_w/M_n calculated by SEC.
[d] Regioregularity (RR) of P3HT calculated by ^{1}H NMR.

Table S2. The 0-0 to 0-1 Peak Intensity Ratio from the Absorption and Emission Spectra of SPNs and PSPNs.

Sample	$A_{0\text{-}0}/A_{0\text{-}1}$	$I_{0\text{-}0}/I_{0\text{-}1}$
SPNs	4.17	1.85
PSPNs	3.45	1.50

The UV-vis spectrum of SPNs (Figure 2A) exhibited a prominent absorption peak at 480 nm, attributed to the π-π* electronic transition of P3HT chains adopting a flexible random-coil conformation. Additionally, a peak appeared at 593 nm, which is indicative of vibronic coupling arising from inter-chain interactions of P3HT thus promoting a higher degree of ordering within the nanoparticles.[38] The spectrum of PSPNs presented similar absorption spectral features, thus indicating that the porosity does not hamper the possibility to use them for optoceutical therapies.[39, 40] The fact that both spectra are similar could also indicate that there are no changes in the chain conformation, in the effective conjugation length of the P3HT segments and in the inter-chain interactions after PLA hydrolysis in PSPNs. The fluorescence emission properties of the nanoparticles upon excitation at 480 nm were also studied (Figure 2B). Both fluorescence spectra, SPNs and PSPNs, showed a maximum emission peak at 634 nm, originated from the 0–0 transition, and a shoulder at 710 nm, attributed to the 0–1 vibronic transition.[41] However, some differences in the exciton bandwidth could be quantitatively estimated from the ratio of the 0–0 transition and 0–1 transition peaks in the absorption and emission spectra (Table S2). A more intense 0-0 emission in relation to the 0-1 emission is indicative of smaller exciton bandwidths and/or increased disorder. In both cases, the ratio $I_{0\text{-}0}/I_{0\text{-}1} > 1$ suggests a structured linear (and coplanar) alignment of thiophene units, characterized by a predominant intrachain coupling and J-aggregate behavior. In the case of PSPNs, the ratio $I_{0\text{-}0}/I_{0\text{-}1}$ experienced a mild decrease, likely due to an expansion in the coherent domain size stemming from the planarization of the more torsionally disordered P3HT chains that results in an increase of the relative interchain coupling, wherein the enhancement in J-coupling outweighs the alterations in H-coupling.[42, 43] Overall, these results prove the occurrence of structural changes in PSPNs in comparison to SPNs, while confirming their main optical responsivity in the visible range.

**Note that reference numbers refer to the reference list of the main manuscript.*

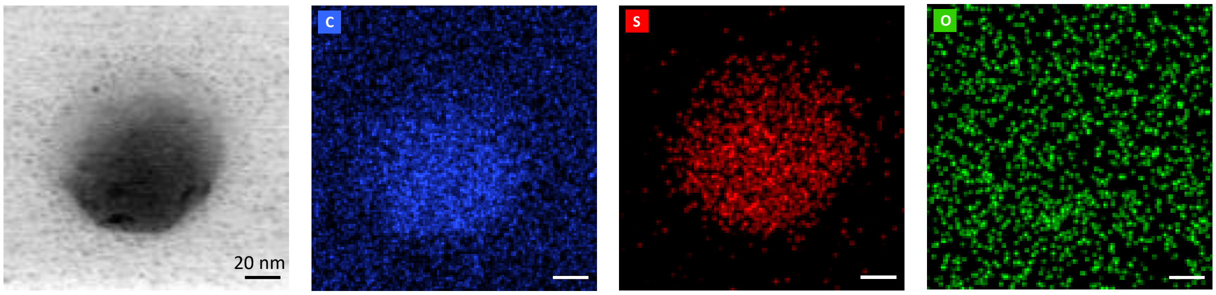

Figure S1. TEM images of non-porous P3HT nanoparticles including the EDX analysis showing sulfur atoms in red, carbon atoms in blue, and oxygen atoms in green. Scale bars = 20 nm.

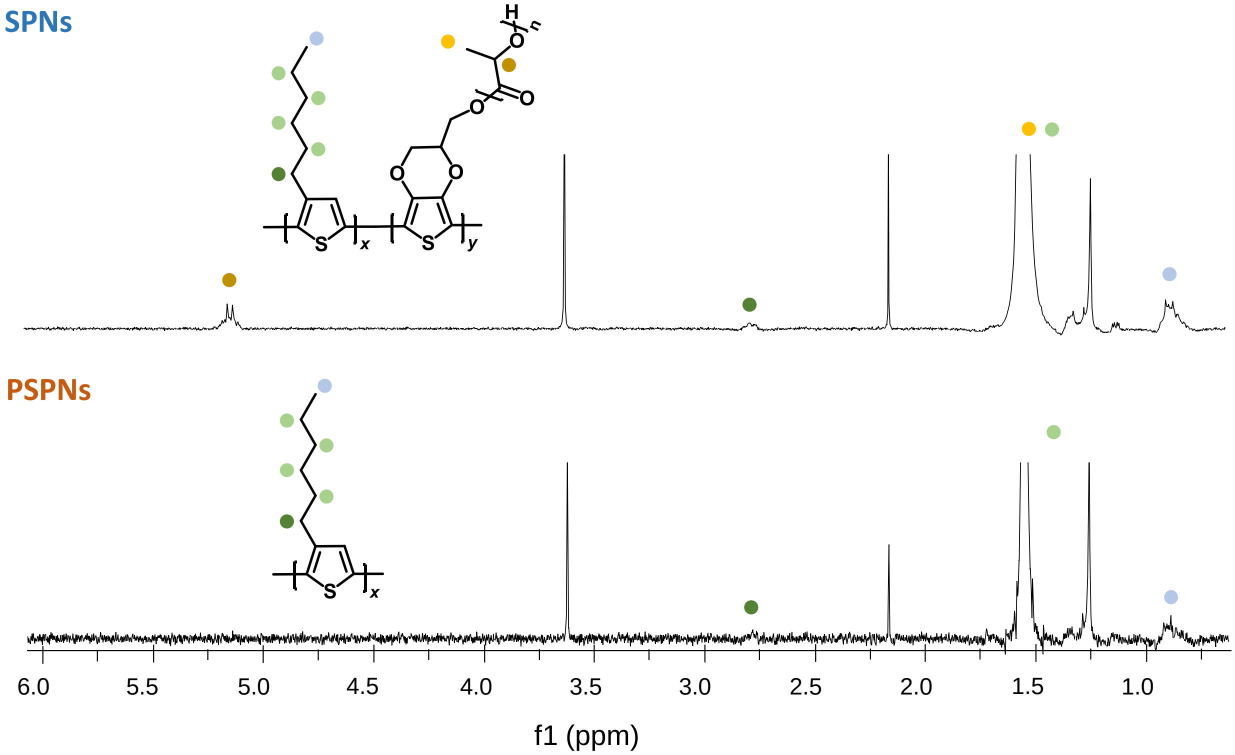

Figure S2. ^{1}H-NMR spectra of SPNs and PSPNs made with the $P3HT_{60}$-*g*-PLA_{40} graft copolymer.

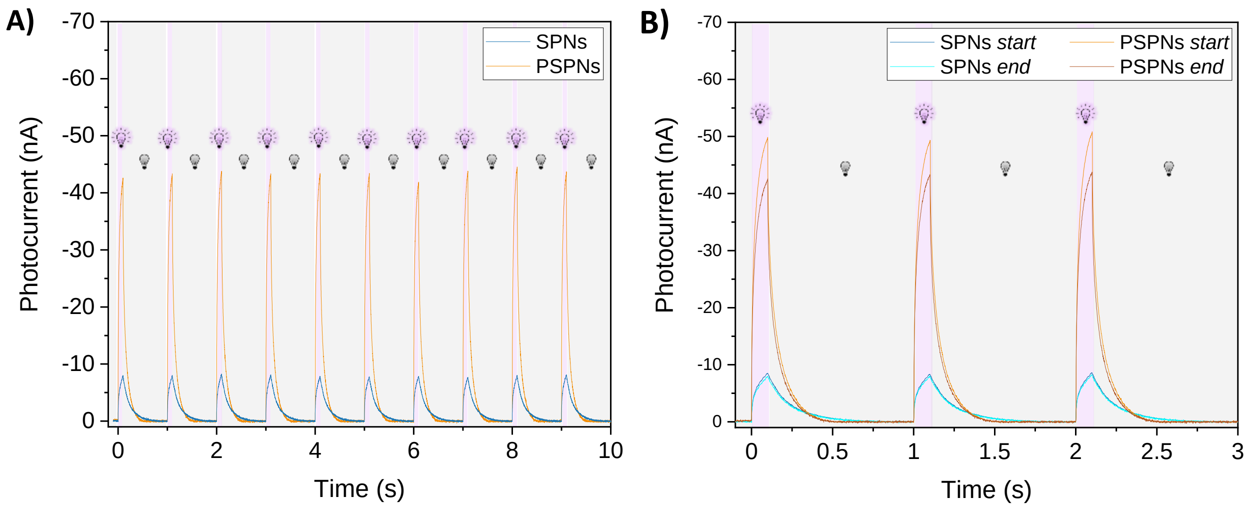

Figure S3. Cyclic photocurrent curves of SPNs and PSPNs in the darkness and irradiated with a LED (λ = 530 nm, 6 mW cm^{-2}, 100 ms ON and 900 ms OFF) mimicking cellular stimulation conditions: A) Last 10 cycles ON-OFF after 6 h cyclic irradiation; B) Overlapping of the first 3 cycles ON-OFF at the beginning of the experiment and the last 3 cycles ON-OFF at the end of the experiment after 6 h irradiation.

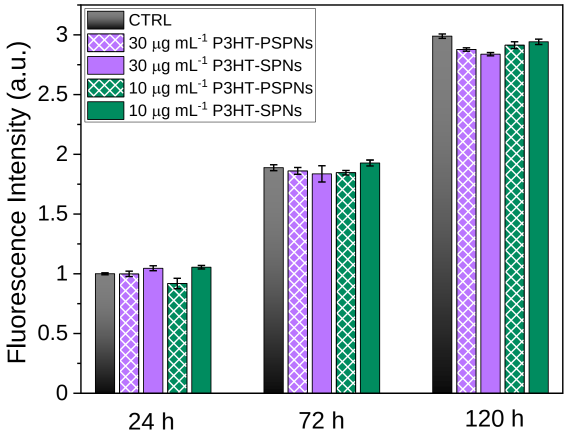

Figure S4. *In vitro* proliferation assay of HUVECs seeded on glass substrates (CTRL) and in contact with different concentrations of SPNs (solid bars) and PSNPs (frame filler bars) up to 120 h.

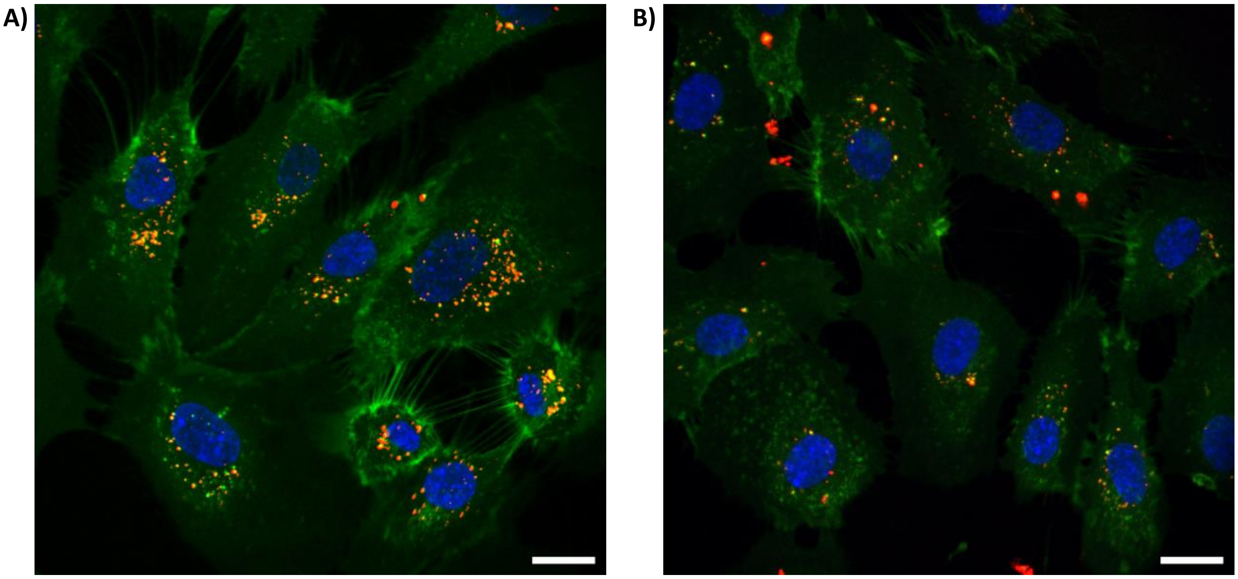

Figure S5. Representative confocal fluorescence images of live HUVECs treated with P3HT-*g*-PLA-SPNs (A) or P3HT-PSPNs (B). The images are acquired at z plane corresponding to the intracellular space. The cells are stained with cell mask green (cell membrane) and DAPI (nuclei). Intrinsic emission of polymer nanoparticles is visible in red. Scale bar, 20 μm.

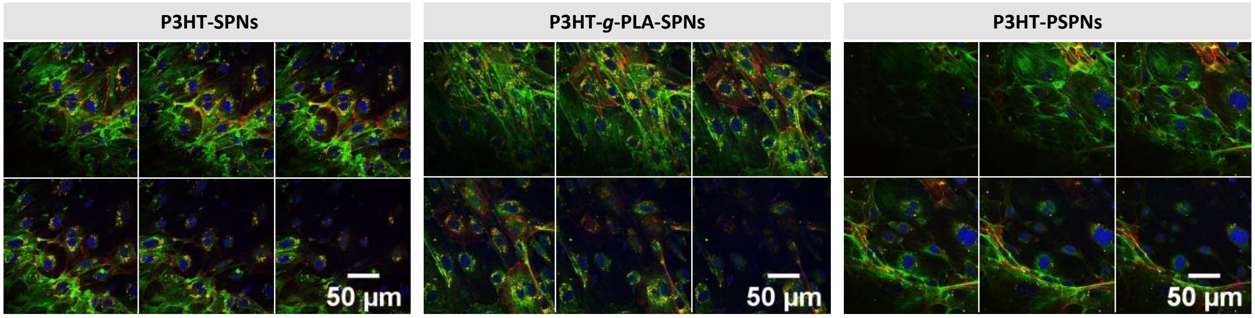

Figure S6. Confocal fluorescence microscopy images of SPNs and PSPNs internalized in HUVECs cells.
